# Fungal fermentation on anaerobic digestate for lipid-based biofuel production

**DOI:** 10.1186/s13068-016-0654-3

**Published:** 2016-11-21

**Authors:** Yuan Zhong, Zhiguo Liu, Christine Isaguirre, Yan Liu, Wei Liao

**Affiliations:** Department of Biosystems and Agricultural Engineering, Anaerobic Digestion Research and Education Center (ADREC), Michigan State University, 524 South Shaw Lane, Room 202, East Lansing, MI 48824 USA

**Keywords:** Anaerobic digestion, Biodiesel, Energy-positive, Fresh-water-free, Fungal fermentation, Lipid, Repeated-batch culture

## Abstract

**Background:**

Anaerobic digestate is the effluent from anaerobic digestion of organic wastes. It contains a significant amount of nutrients and lignocellulosic materials, even though anaerobic digestion consumed a large portion of organic matters in the wastes. Utilizing the nutrients and lignocellulosic materials in the digestate is critical to significantly improve efficiency of anaerobic digestion technology and generate value-added chemical and fuel products from the organic wastes. Therefore, this study focused on developing an integrated process that uses biogas energy to power fungal fermentation and converts remaining carbon sources, nutrients, and water in the digestate into biofuel precursor-lipid.

**Results:**

The process contains two unit operations of anaerobic digestion and digestate utilization. The digestate utilization includes alkali treatment of the mixture feed of solid and liquid digestates, enzymatic hydrolysis for mono-sugar release, overliming detoxification, and fungal fermentation for lipid accumulation. The experimental results conclude that 5 h and 30 °C were the preferred conditions for the overliming detoxification regarding lipid accumulation of the following fungal cultivation. The repeated-batch fungal fermentation enhanced lipid accumulation, which led to a final lipid concentration of 3.16 g/L on the digestate with 10% dry matter. The mass and energy balance analysis further indicates that the digestate had enough water for the process uses and the biogas energy was able to balance the needs of individual unit operations.

**Conclusions:**

A fresh-water-free and energy-positive process of lipid production from anaerobic digestate was achieved by integrating anaerobic digestion and fungal fermentation. The integration addresses the issues that both biofuel industry and waste management encounter—high water and energy demand of biofuel precursor production and few digestate utilization approaches of organic waste treatment.

## Background

Anaerobic digestion (AD) is a biological conversion process that has been proven effective at converting wet organic wastes into biogas, and capable of alleviating the environmental concerns associated with wastes while also producing clean electricity [1–3]. Recently, an increasing number of animal farms are using AD as part of their manure management strategy to produce methane as a renewable energy source [4]. In spite of advantages of renewable energy production and waste confinement, AD only partially utilizes the organic matter in waste streams, particularly for wastes rich in lignocellulosic materials such as dairy/cattle manure and crop residues. The AD effluent (the whole slurry after the digestion) still has relatively high levels of chemical oxygen demand (COD), fiber content, and nutrients (nitrogen and phosphorus). Further treatment is necessary to completely utilize all components in the organic wastes. The AD effluent is commonly separated by a liquid/solid separation unit into two streams: nitrogen- and phosphorus-rich liquid digestate, and fiber-rich solid digestate.

The solid digestate (AD fiber) has been widely recognized by scientific communities as a recalcitrant material that has limited applications such as soil amendment, animal bedding, and plant growing media [5]. Recent studies indicated that compared to other energy crops and agricultural residues, AD fiber demonstrates similar performance as a feedstock for bioethanol and biodiesel production [6–8], which significantly expands the potential application of AD fiber and enhances the economic benefits of anaerobic digestion technology. During the process of AD fiber utilization, chemical pretreatment is required to disrupt the internal association between fiber components and enable saccharification of cellulose and hemicellulose. However, such pretreatment also generates by-products (i.e., furfural, hydroxymethylfurfural, uronic acids, and phenolic compounds) that inhibit the following fermentation process [9, 10]. Our previous studies applied either washing or co-treatment with other feedstocks to reduce the inhibition [6, 8]. In this study, the AD fiber was used as the main feedstock for fermentation, and the washing step was not applied. Instead, a detoxification step is adopted to alleviate the inhibitory effects of the lignocellulose-derived by-products. Many detoxification approaches have been studied to date, such as overliming, reducing agents, polymers, and liquid–liquid extraction [11–14]. Among these approaches, overliming is considered to be as the most effective due to its advantages of less energy consumption, simple operation, and good performance [15, 16]. However, it has also been reported that overliming could cause sugar losses [16]. Therefore, overliming as the selected method for hydrolysate detoxification was investigated in this paper.

The liquid digestate, rich in nitrogen and phosphorus, is commonly used as irrigation water during the growing seasons [17]. However, seasonal demand of such nutrients requires a large storage capability to hold the liquid digestate. In addition, land application of liquid digestate is dependent on crop nutrient demand (nitrogen and phosphorus), which could result in great uncertainty and high cost of liquid digestate handling. To improve economic and environmental sustainability of the liquid digestate handling, alternative approaches are critically needed. Meanwhile, lignocellulosic biorefining demands a significant amount of water for biofuel production, which has triggered concerns about the sustainability of the second-generation biofuel production [18]. Considering the water and nutrient contents in the liquid digestate, if the liquid digestate can be used as the processing water for fermentative biofuel production, it would be an effective solution to address the water demand issue.

Considering the fact that some toxins could remain in the hydrolysate after the detoxification, the working microbes in the following fermentation must be robust enough to withstand them. Our previous studies have demonstrated that the oleaginous fungus, *Mortierella isabellina*, can tolerate relatively high concentrations of toxic compounds in lignocellulosic hydrolysates as well as efficiently consume glucose, xylose, and acetate for lipid accumulation [8, 19–22]. It is apparent that the strain well satisfies the needs of converting anaerobic digestate into value-added fuels and chemicals.

Therefore, this study focused on applying *M. isabellina* to utilize anaerobic digestate (both solid digestate and liquid digestate) for lipid accumulation, and integrating the fungal fermentation with AD to achieve an energy-positive and fresh-water-free process of lignocellulosic biodiesel production. Meanwhile, the studied process also contributes to development of next-generation organic waste management strategies that turn current treatment-based practices into future utilization-based practices. A win–win solution would be thus achieved for both biofuel production and waste management.

## Methods

### Feedstock characteristics

Anaerobic digestion effluent (after the digestion), liquid digestate (after liquid/solid separation), and solid digestate (after liquid/solid separation) were collected from the Michigan State University (MSU) South Campus Anaerobic Digester (42.698800, −84.488068). The digester is a completely stirred tank reactor (CSTR). The effective volume of the digester is 1570 m^3^. The feed of the digester consisted of animal manure from the MSU dairy farm and food wastes from the MSU cafeteria and a nearby food processing facility. The manure and food wastes were mixed at a dry matter ratio of 1.3:2. The characteristics of the mixed feed are listed in Table 1. The digestion temperature and retention time were 40 °C and 25 days, respectively. The biogas is combusted by a 400 kW MAN biogas engine to produce electricity and heat. The digestion performance data are listed in Table 1 as well.

**Table 1:** Characteristics of the feed and performance of the MSU CSTR digester

Characteristics of animal wastes (AD feedstock)	Value
Total solids (%, TS)^a^	8.5 ± 2.2
Volatile solids (%, VS)^a^	7.2 ± 1.9
COD (mg/L)^b^	133,250.0 ± 21,173.0
TP (mg/L)^c^	670.0 ± 6.0
TN (mg/L)^d^	4487.0 ± 788.0

Digester performance	Value
Operating temperature (°C)	40
HRT (days)	25
Biogas production (m^3^/day)	2919.9
Methane composition (% v/v)	60
Mixed wastes feeding the AD (wet tons/day)	76.1
Solid digestate generated (wet tons/day)	11.0
Liquid digestate generated (tons/day)	64.5
Electricity demand for the AD operation (MJ/day)^e^	4593.6
Thermal energy demand for the AD operations (MJ/day)^f^	8161.7

^a^Data is the average of 45 samples with standard deviation

^b^Data is the average of 8 samples with standard deviation

^c^Data is the average of 3 samples with standard deviation

^d^Data is the average of 9 samples with standard deviation

^e^It is for pumps and agitators used in the AD operation

^f^The thermal energy required by the AD unit was calculated by: mass of the wet mixed feed × specific heat of the wet mixed feed × (the digester temperature—the average temperature of the mixed feed) × (1 + 10%), where the average temperature of the mixed feed is 15 °C, the digester temperature is 40 °C, the amount of wet feed is 76,100 kg, the specific heat of the wet mixed feed is 3.9 kJ/kg °C, and 10% was the extra energy that needs to maintain the digester temperature besides heading the feed

After the digestion, a commercial screw press separator with 2 mm screen was used to carry out the liquid/solid separation of the AD effluent. The liquid digestate and solid digestate were obtained accordingly. The characteristics of the AD effluent, liquid digestate, and solid digestate are listed in Table 2.

**Table 2:** Characteristics of the whole, liquid and solid digestates

	AD effluent	Liquid digestate	Solid digestate
Total solids (%)	6.01 ± 0.13	1.83 ± 0.01	30.60 ± 2.13
Volatile solids (% TS)	77.63 ± 0.44	NM^a^	89.18 ± 0.29
COD (mg/L)	41950 ± 4450	17750 ± 354	NM
Carbon (% TS)	42.35 ± 0.54	NM	41.97 ± 0.98
Nitrogen (% TS)	1.48 ± 0.18	NM	1.40 ± 0.02
Total phosphorus (mg/L)	2217.5 ± 12.5	810.0 ± 7.1	NM
Total nitrogen (mg/L)	3075 ± 175	1900 ± 71	NM
Cellulose (% TS)	27.35 ± 0.51	NM	26.52 ± 0.71
Xylan (% TS)	13.82 ± 0.40	NM	13.31 ± 0.65
Lignin (% TS)	31.08 ± 0.76	NM	30.31 ± 0.70
pH (20 °C)	7.18	7.56	NM

Data are the average of three replicates with standard deviation

^a^NM stands for not measured. These NM numbers can be calculated from data of other two streams

### Dilute alkali pretreatment and enzymatic hydrolysis of the digestates

It has been reported that alkali treatment was more efficient to pretreat solid digestate than other treatment methods [23]. Therefore, the solid digestate rich in cellulose, hemicellulose, and lignin was pretreated by a dilute alkali treatment with the conditions of 120 °C for 2 h and a sodium hydroxide (NaOH) concentration of 2% (w/w) using liquid digestate as the processing water. The pretreatment was carried out in 125 mL glass bottles (Wheaton Industries, Millville, NJ), and placed in an autoclave (Brinkmann 2540 M; Tuttnauer USA Co. Ltd., Hauppauge, NY). The effective volume of the slurry in the bottle was 50 mL. The TS of the pretreatment slurry was adjusted to 10% by adding the liquid digestate. After the dilute alkali pretreatment, the pretreated slurry was adjusted to a pH of 5.0 ± 0.2 using 20% (w/w) sulfuric acid (H_2_SO_4_). An enzyme mixture consisting of 9.10 mg cellulase (CTEC 3, protein content: 218 mg/mL; Novozymes North America, Franklinton, NC) and 1.43 mg xylanase (HTEC 3, protein content: 171 mg/mL; Novozymes North America, Franklinton, NC) per gram dry matter of the solid digestate was applied on the pretreated slurry to carry out the enzymatic hydrolysis at 50 °C and 150 rpm (2.5 Hz) in a shaking incubator (Thermo Scientific, Odessa, TX) for 72 h. After the enzymatic hydrolysis, the hydrolysate was centrifuged at 7025*g* for 10 min to separate the liquid hydrolysate from the residual solids. Approximately 2 mL of the liquid hydrolysate was filtered through a 0.22 µm polyethersulfone membrane filter for sugar analysis. The remaining liquid hydrolysate was used for the following detoxification and fermentation tests.

### Overliming detoxification

A completely randomized design (CRD) was adopted to elucidate the effects of overliming conditions (detoxification time and temperature) on sugar recovery and fermentation performance. Three detoxification times (1, 5, and 16 h) and two temperatures (30 and 50 °C) formed six treatments, and each treatment had three replicates, which led to a total of eighteen runs. The detoxification was carried out in 500 mL media bottles (Wheaton Industries, Millville, NJ). Ca(OH)_2_ was added in the liquid hydrolysate until the pH reached 10. The bottles were placed in a shaking incubator (Thermo Scientific, Odessa, TX) at 150 rpm (2.5 Hz) according to the targeted detoxification time and temperature.

After detoxification, the pH of the detoxified hydrolysate was adjusted back to 6 using 20% (w/w) H_2_SO_4_. The detoxified hydrolysate was centrifuged at 7025*g* for 10 min to separate the liquid hydrolysate from the residues. Approximately 2 mL of the hydrolysate was filtered through a 0.22-µm polyethersulfone membrane filter for sugar analysis. The remaining detoxified liquid hydrolysate was stored in the −20 °C freezer for the following fermentation test.

### Oleaginous fungal fermentation

#### Fungal fermentation to evaluate the effects of overliming detoxification

*Mortierella isabellina* ATCC 42613 was used to accumulate lipids on the detoxified hydrolysate medium. The spore and seed cultures were prepared according to methods described by previous studies [20, 24]. The fermentation was carried out in 250 mL Erlenmeyer flasks containing 80 mL of the medium. The detoxified hydrolysate served as the fermentation media. The raw hydrolysate and a synthetic solution containing glucose, xylose, and acetate in concentrations similar to those found in the hydrolysate were used as controls. All media were supplemented with yeast extract (DOT Scientific Inc., Burton, MI) and mineral salts. The yeast extract concentration in the media was 2.0 g/L. The mineral salts in the media were 1 g/L KH_2_PO_4_, 0.5 g/L MgCl_2_·6H_2_O, 0.0014 g/L ZnSO_4_·7H_2_O, 0.0016 g/L MnSO_4_·H_2_O, 0.0036 g/L CoCl_2_·6H_2_O, and 0.00275 g/L FeSO_4_·7H_2_O. All fermentation media were adjusted to pH 6.0 using 20% (w/w) H_2_SO_4_ solution or 30% (w/w) NaOH solution before being sterilized at 121 °C for 15 min. The fermentation medium was inoculated with 10% (v/v) seed and cultivated for 90 h at 25 ± 1 °C on a shaking incubator at 180 rpm (3 Hz).

#### Kinetics of batch fungal fermentation on the selected detoxified hydrolysate

The same dilute alkali pretreatment and enzymatic hydrolysis described in 2.2 was applied on the anaerobic digestate to prepare the hydrolysate. 500 mL flasks containing 200 mL of the digestate slurry were used to carry out the pretreatment and hydrolysis. The selected detoxification method was applied to detoxify the hydrolysate for the batch fungal fermentation. The supplemental nitrogen and mineral salts and inoculation and culture conditions were the same as those described in “Fungal fermentation to evaluate the effects of overliming detoxification” section. The culture was carried out in 500 mL Erlenmeyer flasks containing 200 mL of the medium. The batch fermentation duration was 77 h. Samples were taken along cultivation for sugar, acetate, and fungal biomass analyses. Three replicates were run for this experiment.

#### Kinetics of repeated-batch fungal fermentation on the selected detoxified hydrolysate

The same dilute alkali pretreatment, enzymatic hydrolysis, and detoxification described in “Fungal fermentation to evaluate the effects of overliming detoxification” section were used on the anaerobic digestate to prepare the detoxified hydrolysate for the repeated-batch fungal fermentation. The supplemental organic nitrogen and mineral salts along with inoculation and culture conditions were the same as described in “Fungal fermentation to evaluate the effects of overliming detoxification” section. The culture was carried out in 500 mL Erlenmeyer flasks containing 100 mL of the medium. Once the carbon sources were nearly depleted, the fungal biomass was sterilely separated from the fermentation broth by settling the flasks for 30 min. The supernatant was poured out, and the fresh medium was added to the flasks to carry out the next batch. The operation was repeated twice at 100 and 190 h. Five fermentation broth samples and one fungal biomass sample were collected from each stage for sugar, acetate, biomass and lipid analyses.

### Mass and energy balance

Mass and energy balance analysis was carried out based on 1000 kg dry mixed feed for the anaerobic digestion. The target system included both anaerobic digestion and fungal fermentation of lipid accumulation. The performance data of the MSU CSTR digester were used to conclude the mass and energy balance of the anaerobic digestion operation. The data from the aforementioned hydrolysis, detoxification, and fermentation experiments were used for the mass and energy balance of the lipid production on the digestate according to a reported method [25].

### Analytical methods

COD, TP, and TN were determined using HACH kits (product number: 2125915, 2767245 and 2714100, HACH Company, Loveland, CO). TS and VS were analyzed according to APHA [26]. Elemental analysis of the AD fiber was conducted using an element analyzer (ECS 4010, Costech Analytical Technologies Inc., Valencia, CA) in the MSU Soil Biology Lab. Fiber composition of the solid digestate was measured following the Laboratory Analytical Procedure (LAP) developed by the National Renewable Energy Laboratory (NREL) [27].

Glucose, xylose, and acetate in the hydrolysate and fermentation media were determined by high performance liquid chromatography (HPLC) (Shimadzu Corp., Kyoto, Japan) equipped with an analytical column (Aminex HPX-87H, Bio-Rad Laboratories, Inc., Hercules, CA) and a refractive index detector (Shimadzu Corp., Kyoto, Japan). The mobile phase was 0.005 mol/L sulfuric acid at a flow rate of 0.6 mL/min. The oven temperature was set at 65 °C. HPLC-grade standards including glucose, xylose, and sodium acetate were purchased from Sigma (Sigma–Aldrich, St. Louis, MO).

Fungal biomass was collected by filtration and washed three times with deionized water. Cell biomass was determined by drying at 105 °C until a constant weight was achieved. The dried cell biomass was ground in a mortar for lipid extraction. Total lipid was determined gravimetrically [28].

### Statistical analysis

A two-way ANOVA using the Statistical Analysis System 9.0 (SAS Institute Inc., Cary, NC) was carried out to analyze the significance of impacts of two detoxification factors on sugar recovery and fermentation performance. A pair-wise comparison using SAS 9.0 was also conducted to identify significant differences between experimental runs.

## Results and discussion

### Characteristics of AD effluent, solid digestate, and liquid digestate

The characteristics of the AD effluent, solid digestate, and liquid digestate indicate that the solid digestate was the only stream that has both desirable TS content (30.60%) and carbohydrate contents (26, 13, and 30% of cellulose, xylan, and lignin, respectively) to be used as the lignocellulosic feedstock for fungal lipid accumulation (Table 2). In addition, the liquid digestate contained a significant amount of nitrogen and phosphorus (1900 and 810 mg/L, respectively), which represents a potential nutrient stream to support the fungal fermentation. Liquid and solid digestates can be mixed to achieve desirable TS, carbohydrate, and nutrient contents for different microbial processes. In this study, the TS content of the mixture feed was set at 10% to carry out pretreatment, hydrolysis, and fermentation based on previous *M. isabellina* studies on lignocellulosic materials [8, 20, 29].

### Effects of overliming detoxification on sugar recovery and fungal fermentation

**Fig. 1:**
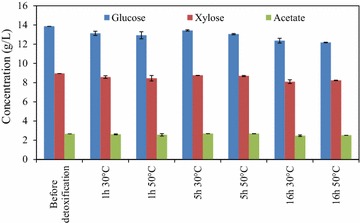
Effects of detoxification on sugar and acetate concentrations*. *: Data are the average of three replicates with standard deviation

After pretreatment and hydrolysis, the mixture feed at the TS of 10% generated a hydrolysate containing 13.85 g/L of glucose, 8.95 g/L of xylose, and 2.67 g/L of acetate. The effects of different detoxification conditions on sugar and acetate concentrations in the hydrolysate are demonstrated in Fig. 1. The pair-wise comparison concluded that there were no significant (*P* > 0.05) differences on sugar and acetate concentrations between non-detoxified and detoxified hydrolysates. This result indicates that the tested overliming conditions did not lead to significant substrate loss in hydrolysates, which was different from other overliming treatment studies on hydrolysates from bagasse and other lignocellulosic materials [16, 30].

**Fig. 2:**
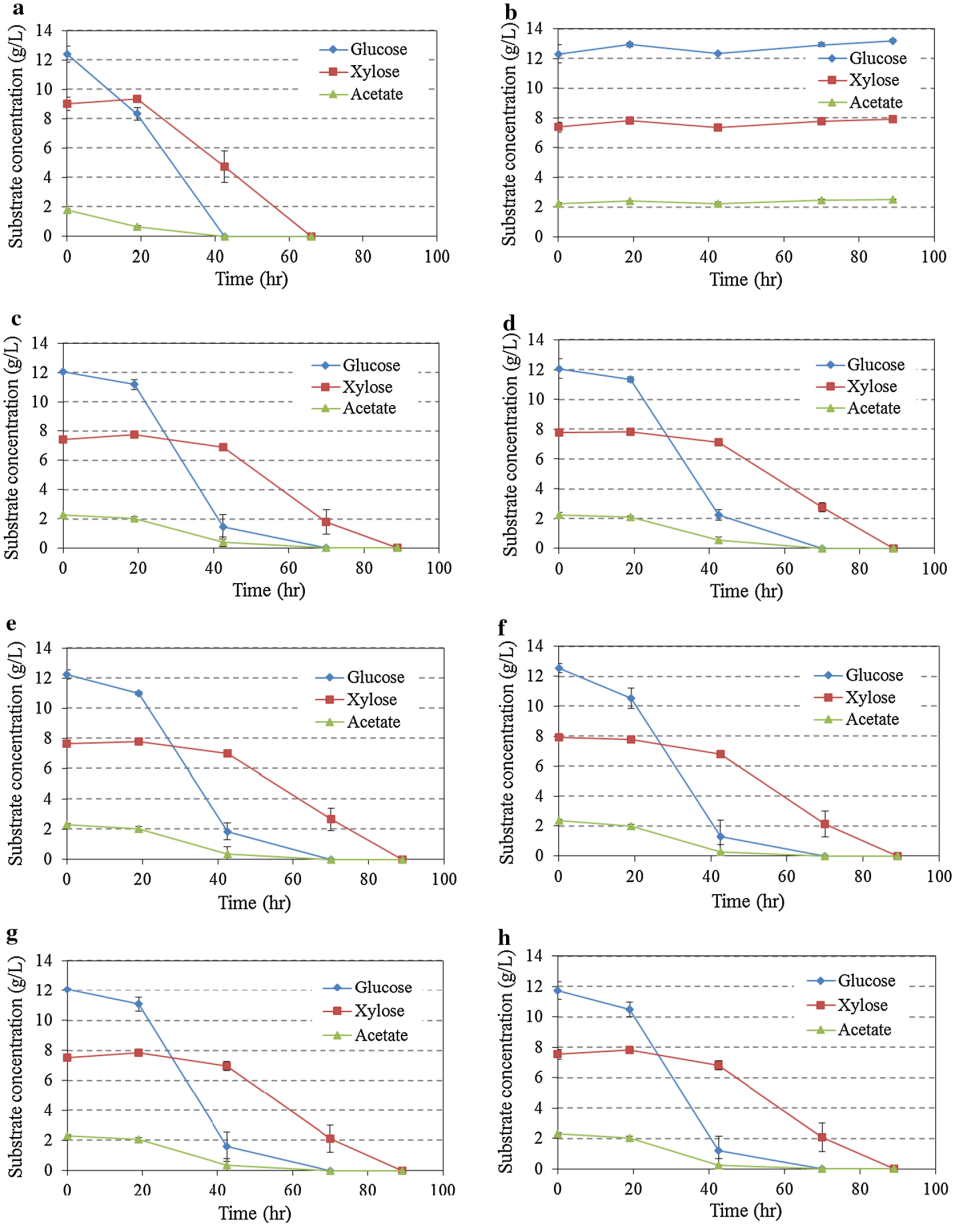
Effects of detoxification on fungal fermentation*. *: Data are the average of three replicates with standard deviation. **a** Synthetic medium; **b** without detoxification; **c** 1 h 30C; **d** 1h 50C; **e** 5h 30C; **f** 5h 50C; **g** 16h 30C; **h** 16h 50C

To further evaluate effects of the overliming on fungal fermentation, the hydrolysates from different detoxification conditions were used to carry out *M. isabellina* cultivation. The substrate consumption of *M. isabellina* on the hydrolysates are presented in Fig. 2. Sugars and acetate were not consumed in the hydrolysate without detoxification during the 89 h culture period (Fig. 2b). The lack of sugar consumption clearly demonstrates a strong inhibitory effect of the alkali pretreated hydrolysate on *M. isabellina*. Detoxification is necessary to make the hydrolysate amendable to the fungal strain. With overliming detoxification, the sugars and acetate were completely consumed in 89 h of fermentation for all hydrolysates under different overliming conditions, which indicates that the detoxification did effectively alleviate the inhibitory effect of the hydrolysates on the fungal strain. However, compared to the culture on the synthetic medium (all sugars and acetate were consumed in 66 h), a delay (23 h) of substrate consumption was observed from the cultures on detoxified hydrolysates (Fig. 2c–h).

To determine the preferred detoxification condition, lipid production was used to statistically compare the effects of detoxification conditions on fungal fermentation (Table 3). *M. isabellina* on the synthetic medium consumed all substrates and generated 3.69 g/L lipid. Compared to the culture on the synthetic medium, the cultures on the detoxified hydrolysates overall produced less lipid. The accumulated lipid and lipid productivities of the cultures on the detoxified hydrolysates ranged from 1.52 to 3.48 g/L and from 0.41 to 0.94 g/L/d, respectively. The corresponding lipid-substrate conversion were between 0.07 and 0.16 g/g. The two-way ANOVA elucidates that both detoxification temperature and time did significantly (*P* < 0.05) influence the lipid concentration, lipid-substrate conversion, and lipid productivity. The pair-wise comparison concludes that the detoxification conditions of 30 °C and 5 h had significantly (*P* < 0.05) better performance on fungal lipid accumulation (Table 3), in which lipid concentration, lipid-substrate conversion, and lipid productivity reached 3.48 g/L, 0.16 g/g, and 0.94 g/L/d, respectively. Therefore, 30 °C and 5 h was selected as the preferred detoxification method.

**Table 3:** Fungal lipid production on the hydrolysates from different detoxification conditions

	Synthetic medium	30 °C detoxification			50 °C detoxification		
		1 h	5 h	16 h	1 h	5 h	16 h
Lipid concentration (g/L)	3.69 ± 0.11	2.17 ± 0.48	3.48 ± 0.05	2.07 ± 0.13	2.81 ± 0.70	2.07 ± 0.13	1.52 ± 0.23
Lipid-substrate conversion (g/g)	0.16 ± 0.01	0.10 ± 0.02	0.16 ± 0.01	0.08 ± 0.01	0.13 ± 0.03	0.09 ± 0.00	0.07 ± 0.01
Lipid productivity (g/L/d)	1.34 ± 0.04	0.58 ± 0.13	0.94 ± 0.01	0.46 ± 0.05	0.76 ± 0.19	0.56 ± 0.03	0.41 ± 0.06

Data are the average of three replicates with standard deviation

### Fungal lipid accumulation on the selected detoxified hydrolysate

**Fig. 3:**
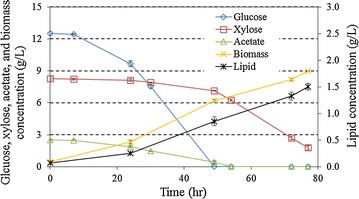
Batch fungal fermentation on the selected detoxified hydrolysate*. *: Data are the average of three replicates with standard deviation

The effect of the preferred detoxification on fungal fermentation of biomass and lipid accumulation was then investigated. A batch fungal fermentation on the selected detoxified hydrolysate (under the detoxification conditions of 5 h and 30 °C) was performed (Fig. 3). The substrate consumption kinetics were very similar to the cultures in “Effects of overliming detoxification on sugar recovery and fungal fermentation” section. The glucose and acetate were completely consumed in 49–54 h, respectively. At the end of batch culture (77 h), 1.79 g/L xylose remained in the broth, and 8.98 g/L biomass and 1.50 g/L lipid were accumulated. The corresponding lipid and biomass yields were 0.07 and 0.42 g/g, respectively (Table 4).

**Fig. 4:**
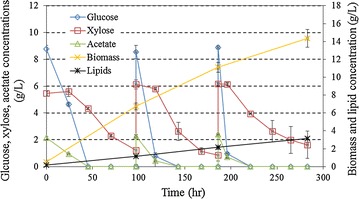
Repeated-batch fungal fermentation on the selected detoxified hydrolysate*. *: Data are the average of three replicates with standard deviation

Due to relatively low carbohydrate content (26.52% cellulose and 13.31% xylan) in the solid digestate (Table 2), the biomass and lipid concentrations were relatively low from the batch culture. Lipid concentration is a very important parameter for downstream separation and extraction as well as the process economics, thus methods for improvement should be investigated. Since lipid is a structural component of fungal biomass, accumulating more biomass substantially and directly leads to higher lipid concentration. Therefore, a repeated-batch fermentation culture, a common approach to enhance the biomass accumulation [31, 32], was adopted to improve the fermentation performance of fungal biomass and lipid production. Kinetics of the repeated-batch fermentation is shown in Fig. 4. The trends of substrate consumption, fungal biomass, and lipid accumulation were similar to the batch culture; however, the substrate consumption rates dramatically increased from the initial batch to the 2nd repeated batch, and so on. The glucose consumption rates were 4.68, 9.32 and 19.37 g/L/d, the corresponding acetate consumption rates were 1.15, 1.84, and 4.06 g/L/d, and the xylose consumption rates were 0.77, 1.04, and 1.12 g/L/d in the initial batch, 1st repeated-batch, and 2nd repeated-batch, respectively. The substantial increase in the substrate consumption rate in subsequent stages of fermentation may be explained by continuous accumulation of the fungal biomass. Higher biomass concentration in the late stages demands more nutrients, which leads to a higher substrate consumption rate. In addition, it is also possible that the fungal strain was gradually ‘trained’ during the repeated-batch fermentation to adapt to the ‘harsher’ culture environment so that the lag time was significantly shortened in the later stages. Further investigations are needed to draw an accurate conclusion.

Biomass and lipid accumulation kinetics also demonstrate that the biomass concentrations significantly increased from 6.74 g/L in the initial batch to 11.11 g/L in the 1st repeated-batch and 14.34 g/L in the 2nd repeated-batch (Fig. 4; Table 4). The lipid concentrations correspondingly changed from 1.25 to 2.17 to 3.16 g/L (Table 4). The lipid contents in the biomass also increased from 0.17 to 0.20 to 0.21 g/g while the lipid yields remained at approximately 0.07 g/g for all three stages during the repeated-batch culture. The biomass yields were reduced from 0.45 to 0.36 to 0.31 g/g. An increase in metabolism in the later stages of the culture, leading to more substrate oxidation (generating energy and carbon dioxide) than substrate assimilation (accumulating biomass and metabolites), may provide an explanation for the decrease in biomass yields over the course of cultivation. At the end of the repeated-batch fermentation, the biomass and lipid concentrations reached 14.36 and 3.16 g/L, respectively, which were 1.60 and 2.11 times more than the corresponding concentrations (8.98 and 1.50 g/L) from the batch culture (Table 4).

**Table 4:** Fermentation performance of batch and repeated-batch cultures

Culture	Batch (control)	Repeated-batch		
		Initial batch	After 1st repeated-batch	After 2nd repeated-batch
C/N ratio of the medium (mol/mol)	3.07 ± 0.04	2.16 ± 0.06	2.16 ± 0.06	2.16 ± 0.06
Glucose consumed (g/L)	12.51 ± 0.17	8.77 ± 0.26	17.31 ± 0.50	26.23 ± 0.45
Xylose consumed (g/L)	6.47 ± 0.08	4.14 ± 1.17	9.06 ± 0.34	12.77 ± 0.50
Acetate consumed (g/L)	2.51 ± 0.04	2.15 ± 0.11	4.42 ± 0.12	6.83 ± 0.08
Biomass concentration (g/L)	8.98 ± 0.04	6.74 ± 0.39	11.11 ± 0.54	14.34 ± 0.99
Lipid concentration (g/L)	1.50 ± 0.06	1.25 ± 0.06	2.17 ± 0.06	3.16 ± 0.08
Lipid content in biomass (g/g)	0.17 ± 0.01	0.17 ± 0.01	0.20 ± 0.01	0.21 ± 0.00
Lipid yield (g/g)^a^	0.07 ± 0.00	0.08 ± 0.00	0.07 ± 0.00	0.07 ± 0.00
Biomass yield (g/g)^b^	0.42 ± 0.01	0.45 ± 0.04	0.36 ± 0.02	0.31 ± 0.01

Data are the average of three replicates with standard deviation

^a^Lipid yield is with respect to the total amount of substrates in the medium

^b^Biomass yield is with respect to the total amount of substrates in the medium

Even though the repeated-batch culture significantly enhanced biomass and lipid production, lipid yields were still lower compared to the previous studies using lignocellulosic materials [8, 19, 20, 33]. Zhong et al. reported a lipid yield of 0.15 g/g cultured on the hydrolysate from a mixture of corn stover and solid digestate [8]. Ruan et al. reported lipid yields of 0.12, 0.15, 0.13, and 0.10 g/g on the hydrolysates from corn stover, switchgrass, miscanthus, and giant reed, respectively [20]. The low lipid yield in this study was probably caused by the low C/N ratio. The C/N ratios of the hydrolysates used for the fungal cultivation ranged from 2.16 to 3.07 (Table 4), which were several magnitudes lower than the reported ratios. It has been reported that oleaginous organisms tend to accumulate more lipids under nitrogen limited culture conditions [24, 34]. The typical C/N mole ratios used for *M. isabellina* culture of lipid accumulation were in the range of 60–300 [24]. Therefore, it is apparent that adjusting C/N ratio of the hydrolysate from digestates is critical to further improve fungal lipid yield.

### Mass and energy balance analysis

**Fig. 5:**
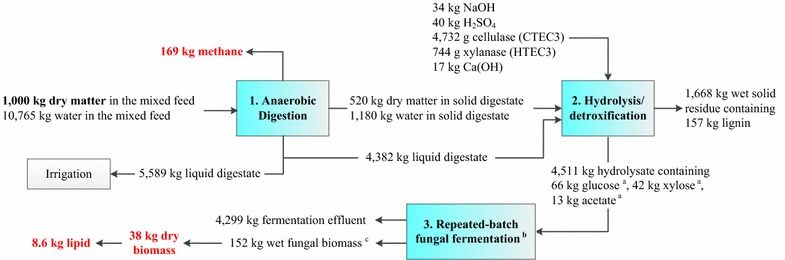
Mass balance of the fungal lipid production on anaerobic digestate including both anaerobic digestion and fungal fermentation. *a* The glucose, xylose, and acetate concentrations of 13.85, 8.95 and 2.67 g/L were used for the calculation. *b* The biomass and lipid conversion (0.31 and 0.07 g/g substrates) from the repeated-batch operation were used to calculate the fungal biomass and lipid production. *c* The dry matter of the wet biomass was 25%

A mass and energy balance was conducted to evaluate the system performance (Fig. 5). The AD generates 169 kg methane, 1700 kg wet solid digestate, and 9971 kg liquid digestate per 1000 kg dry mixed feed to the digester (Fig. 4). A portion of the liquid digestate (4382 kg) was mixed with 1700 kg wet solid digestate to prepare the anaerobic digestate with 10% TS for hydrolysis/detoxification and fungal fermentation. The remaining liquid digestate (5589 kg/1000 kg dry mixed feed) rich in nitrogen and phosphorus can be used as a liquid fertilizer for crop production. The hydrolysis and detoxification applying three unit operations of NaOH pretreatment, enzymatic hydrolysis, and Ca(OH)_2_ detoxification generated a hydrolysate (4511 kg) that contained 66 kg glucose, 42 kg xylose, and 13 kg acetate. The hydrolysis and detoxification also generated 1668 kg wet solid residue rich in lignin (containing 157 kg lignin). The wet solid residue after drying can be used as the solid fuel for energy production. The repeated-batch fungal fermentation on the hydrolysate produced 38 kg dry fungal biomass with 23% lipid. The mass balance results also demonstrate that the liquid digestate provided enough water to satisfy the water demand of the process, which addresses one of the major challenges—a large amount of fresh water needed for fermentation processes of biofuel and chemical production [35–38].

Energy balance shows that integrating AD and fungal fermentation leads to an energy-positive system of fungal lipid production (Table 5). Based on 1000 kg dry mixed feed, AD as a power unit in the system generated a net energy of 6478 MJ. The hydrolysis and detoxification operation consumed 1593 MJ. The hydrolysis residue rich in lignin can produce 3830 MJ, which led to a positive energy output of 2237 MJ for the hydrolysis and detoxification operation. The fungal fermentation as an aerobic cultivation process was the most energy-intensive unit operation in the system. It required 9048 MJ to complete the fermentation. 8.6 kg lipid from 38 kg dry fungal biomass had an energy content of 344 MJ. Due to energy production from biogas and lignin, a positive overall net energy of 11.09 MJ was achieved by the integrated system.

**Table 5:** Energy balance of the fungal lipid production on anaerobic digestate

Energy balance^a^	AD	Hydrolysis/detoxification^b^	Fungal fermentation^c^
Energy input (MJ/1000 kg dry mixed feed)	−1971.96^d^	−1593.48^e^	−9048.27^f^
Energy output (MJ/1000 kg dry mixed feed)	8450.00^g^	3830.80^h^	344.00^i^
Net energy (MJ/1000 kg dry mixed feed)	6478.04	2237.32	−8704.27
Overall net energy (MJ/1000 kg dry mixed feed)	11.09		

All inputs are assigned “−”, and all outputs are assigned “+”. The system includes AD, hydrolysis/detoxification, and fungal fermentation (Fig. 5)

^a^Data were calculated and adjusted based on 1000 kg dry mixed feed

^b^Hydrolysis/detoxification includes unit operations of pretreatment, enzymatic hydrolysis, and detoxification

^c^The fungal fermentation includes unit operations of fungal fermentation and biomass drying

^d^The energy input for the AD unit includes both heat and electricity, which is calculated from the data in Table 1

^e^Compared to the pretreatment and enzymatic hydrolysis, the energy demand for the detoxification (under 50 °C and 5 h) is negligible. Thus, the energy consumption of 262 MJ/m^3^ reaction solution for the pretreatment and enzymatic hydrolysis was used for the calculation [25]. The energy input of the hydrolysis/detoxification is 1593.48 MJ/1000 kg dry mixed feed

^f^The energy input of the fungal fermentation operation is 10,286.67 MJ/1000 kg dry mixed feed according to the reference numbers of 1938.77 MJ/m^3^ fermentation broth and 1.99 MJ/kg wet biomass [25]. The amount of the fermentation broth is 4511 kg. The amount of the wet fungal biomass is 152 kg

^g^The energy output of the AD is the methane energy. Low heating value of methane of 50 MJ/kg methane was used for the calculation

^h^The lignin has a low heating value of 24.4 MJ/kg lignin. 157 kg lignin/1000 kg mixed feed was generated from the process

^i^The lipid as the product is used for biodiesel production. The lipid has a low heating value of 40.0 MJ/kg lipid

Based on the mass and energy balance analysis, a self-sustaining process to produce lipid from anaerobic digestate has been realized by combining AD and aerobic fungal fermentation. The results also indicate that improving hydrolysis (the current conversion of cellulose and xylan to glucose and xylose were only 47 and 64%, respectively) and fermentation (the lipid yield was only 0.07 g per g substrates) efficiencies could significantly enhance the system performance of lipid production. The related studies are currently being carried out in the authors’ labs.

## Conclusions

A fresh-water-free and energy-positive process was developed in this study to simultaneously utilize both solid and liquid digestates to accumulate fungal lipids for biodiesel production. The mixture of solid and liquid digestates was pretreated and hydrolyzed by alkali and enzyme, respectively, to release mono-sugars. The subsequent overliming detoxification process was then applied to prepare the hydrolysate for fungal fermentation. The repeated-batch fungal cultivation significantly increased biomass and lipid concentrations. The methane production in the AD operation provided the energy for the fungal lipid accumulation on the digestate, which leads to an energy-positive process using aerobic fermentation for production of fuel precursor. The results clearly demonstrate that the studied process has potential to be a win–win solution for both biofuel production and waste management.

## Abbreviations

AD: anaerobic digestion
COD: chemical oxygen demand
CSTR: completely stirred tank reactor
TS: total solids
CRD: completely randomized design
TP: total phosphorus
TN: total nitrogen
C/N: carbon:nitrogen
